# Appropriate Therapeutic Disclosures in Improving Client Engagement in Mental Health Management

**DOI:** 10.1192/j.eurpsy.2023.2103

**Published:** 2023-07-19

**Authors:** L. Myers

**Affiliations:** 1 Private, Sydney, Australia

## Abstract

**Introduction:**

As psychiatrists, we are taught not to disclose and to present a blank canvas to the client

Should mental health professionals be reconsidering this stance if aiming to effectively manage their clients who live in a world that promotes vulnerability and lived experience as powerful therapeutic strategies?

**Objectives:**

Promote mental health and remove the shame and stigma limiting client engagement by advocating for ‘real’ psychiatrists

**Methods:**

- Discuss therapeutic disclosure and its history
- The impact of social media and current trends in mental health promotion
- Suggestions for improving client engagement through reclaiming the expert role in mental health promotion and the value of sharing lived experience within professional boundaries. To

**Results:**

- Clients improve communication and openness
- Mental health engagement promoted and encouraged

**Conclusions:**

Clients respond favourably to psychiatrist vulnerability and authenticity with therapeutic disclosures

**Disclosure of Interest:**

None Declared

